# Core outcome set for clinical studies of postoperative ileus after intestinal surgery

**DOI:** 10.1093/bjs/znac052

**Published:** 2022-03-08

**Authors:** Tripartite Gastrointestinal Recovery Post-operative IIeus Group, S J Chapman, M J Lee, S Blackwell, R Arnott, R P G ten Broek, C P Delaney, N N Dudi-Venkata, R Fish, D Hind, D G Jayne, K Mellor, A Mishra, G O’Grady, T Sammour, G Thorpe, C I Wells, A M Wolthuis, N S Fearnhead, S Adegbola, G Bagaglini, M Bath, N Bibby, C Bisset, N Blefari, N S Blencowe, W Bolton, J P Bulte, J Burch, M Campanelli, O Cano-Valderrama, J Carver, C Challand, S Chan, S Chandler, D Clerc, P Coe, D Cox, K L R Cross, A Culkin, V Cuthill, S Daniels, A Dawson, L Dawson, F Dixon, C Downey, T Drake, S Duff, G Dunning, E Espin-Basany, M D Evans, M Fakhrul-Aldeen, N Fisher, S Fleetwood-Beresford, G Gallo, Z Garoufalia, R George, J Han, D Harji, R Harmston, D A Harris, M Mohammed, J Helliwell, J Hepburn, P Herrod, N Horwood, C Keane, S Kelly, H M Kroon, M D S Lonsdale, G Major, J Mattison, A Lawson McLean, M Millan, S Limbert, F McDermott, A Mehraj, C Moriarty, S Moug, E Murray, M Naylor, D Nepogodiev, J Oliver, D Pandey, F Pata, H M Paterson, A Peckham-Cooper, G Pellino, P Pockney, V K Proctor, D Proud, V Rew, M Rutegård, K Sahnan, A Sayers, L Siragusa, R W Smillie, J Spratt, D Swain, S Taylor, P Tejedor, O Thomas, J Thompson, K Tsimogiannis, D Tuohey, R Vissapragada, M U Younis, P G Vaughan-Shaw, K Whyte, K Wheelband, A Williams, A Yates, R Young

**Affiliations:** Leeds Institute of Medical Research at St James’s, University of Leeds, Leeds, UK

## Introduction

Postoperative ileus is a common and distressing complication after intestinal surgery^1^. It presents clinically as impairment of intestinal motility, characterized by abdominal pain, vomiting, and delayed recovery of defaecatory function. For patients, this increases the risk of serious complications, such as pneumonia, venous thromboembolic events, and malnutrition^2^. For healthcare systems, it leads to a substantial economic burden associated with increased medical, nursing, dietitian, and laboratory costs^3^. Accordingly, postoperative ileus is now recognized as a research priority by expert and public stakeholder groups^4^.

Numerous clinical interventions have been evaluated in efforts to prevent postoperative ileus, but few have led to meaningful patient benefit^5^. A key challenge for researchers is the absence of a standardized and agreed framework to describe the effectiveness of new interventions in clinical studies^6^. Common outcomes include the time taken until first passage of flatus/stool, time until tolerance of oral diet, and the return of bowel sounds. It remains unclear, however, whether these are sufficiently relevant to patients and healthcare professionals when evaluating new treatments and implementing them in clinical practice^7^.

A solution to this problem is the development of an agreed core outcome set developed through patient–clinician consensus. Core outcome sets provide a minimum set of outcomes that should be reported in all studies of a defined clinical condition and are supported by the Core Outcome Measures in Effective Trials (COMET) Initiative^8^. The present report describes the international development and final content of an agreed core outcome set for postoperative ileus relevant to patients undergoing intestinal surgery.

## Methods

### Ethics and governance

Research ethics approval was confirmed by the University of Sheffield Ethics Committee on 27 September 2019. A collaborative steering committee was convened with representation from Asia, Australasia, Europe, and North America, and included medical, allied healthcare professional, and patient investigators. The study was registered with the COMET Initiative and the protocol was reported previously^9,10^. An extended description of the methods and results is provided in *Appendix S1*. An abridged summary is reported here.

### Scope and definitions

The scope of the core outcome set was defined according to the Core Outcome Set—Standards for Development recommendations^11^. The health condition was postoperative ileus; the population was adult patients undergoing intestinal surgery for any indication; and the setting was clinical studies assessing the effectiveness of a clinical intervention to reduce ileus. Intestinal surgery was considered to represent any intra-abdominal procedure via any surgical approach on the intestinal tract with or without formation of a stoma.

### Participants

Stakeholder representation was designed to reflect the multidisciplinary management of postoperative ileus as well as the challenge it presents on an international scale. Three key stakeholder panels were defined: patients with previous experience of intestinal surgery, allied healthcare professionals (including nurses and dietitians), and medical professionals (including abdominal surgeons and perioperative clinicians). Medical and allied healthcare professionals were considered as two separate panels throughout the study to ensure that potentially diverging perspectives were captured. During each phase of the study, participants were recruited via national and international organizations as well as through social media.

### Overview of study methods

The study consisted of three phases, in accordance with Delphi methodology, and was conducted between 17 January 2020 and 6 March 2021^10^. In phase 1, a long list of candidate outcomes was generated from a systematic review of previous literature, a series of four international patient and clinician focus groups, and consultation within the steering committee^12^. In phase 2, candidate outcomes were presented to stakeholders via a three-round Delphi survey with between-round feedback. Suggestions for additional outcomes were invited during round 1. During each round, participants voted on the importance of each outcome using a numerical rating scale (1–9), and those that fulfilled a predefined threshold of consensus were carried forward iteratively to the consensus meeting (*Table 1*). In phase 3, an online consensus meeting was convened to ratify the final outcome set. This was chaired by an independent chairperson and participants were sampled purposively to represent key stakeholder groups across an international setting. Final anonymized voting took place on the final composition and presentation of the set.

**Table 1:** Criteria for consensus

	Criteria
**Delphi process**	Consensus was achieved if: ≥ 70% of participants from each stakeholder group rated an outcome between 7 and 9 on the numerical rating scale *or* ≥ 90% of participants from a single stakeholder group rated an outcome between 7–9 on the numerical rating scale
	An extended threshold was set for consideration of ‘borderline’ outcomes: ≥ 65% of participants from each stakeholder group rated an outcome between 7 and 9 on the numerical rating scale during round 3 of the Delphi process
**Consensus meeting**	Decisions were ratified if: ≥ 80% of participants voted in favour of the proposed consensus statement

### Patient and public involvement

Two patient representatives joined the steering committee, and contributed to the design, delivery (including the provision of plain English versions of outcomes; *Table S1*), analysis, and decision-making throughout. They encouraged patient engagement during the Delphi process and ensured that the patient voice remained central.

## Results

### Participants

A total of 155 participants took part in round 1 of the Delphi survey (155 of 234, 66.3 per cent). After completing round 1, 123 of 155 (79.4 per cent) took part in round 2, and 112 of 123 (91.1 per cent) in round 3. There were 15 participants in the final consensus meeting, including five patients, two allied healthcare professionals (1 nurse and 1 dietitian), and eight medical professionals (*Table 2*).

**Table 2:** Participant characteristics of Delphi and consensus meeting stages

	Round 1 Delphi (*n* = 155)	Round 2 Delphi (*n* = 123)	Round 3 Delphi (*n* = 112)	Consensus (*n* = 15)
**Stakeholder group**				
Patients	41	33	29	5
Allied healthcare professionals*	21	14	12	2
Medical professionals	93	76	71	8
**Location**				
Asia	3	3	3	1
Africa	2	2	1	0
Australasia	24	14	13	3
Europe (non-UK)	20	16	16	2
North America	1	1	1	0
UK	105	87	78	9

* Breakdown of Allied Healthcare Professionals: Round 1 Delphi—8 dietitians, 13 nurses; round 2 Delphi—5 dietitians, 9 nurses: round 3 Delphi—5 dietitians, 7 nurses; consensus meeting—0 dietitians, 2 nurses.

### Outcome longlisting

Seventy-three outcomes were identified from a systematic review of previous evidence which was refined by the steering committee to eliminate duplication (*Fig. S1*)^12^. Six unique outcomes were added from stakeholder focus groups and 12 by the steering committee, resulting in 75 unique outcomes used to populate round 1 of the Delphi process.

### Delphi process

During round 1, a total of 75 outcomes were presented to participants and 13 reached the threshold to be considered at the consensus meeting. Eight unique outcomes were generated via free-text responses and were carried forward to round 2 (*Box S1*). During round 2, 70 outcomes (including those generated in the earlier round) were presented and nine reached the meeting threshold. The remaining 61 outcomes were re-presented during round 3 and one more reached the threshold. After consideration by the steering committee, six outcomes were considered to have ‘borderline’ consensus, and it was agreed to re-present these for a final decision at the meeting. The full Delphi results are shown in *Tables S2* and *S3*.

### Consensus meeting

Twenty-nine outcomes were considered by participants during the consensus meeting. Following detailed discussion, 23 outcomes that had achieved consensus during the Delphi process were ratified and two of six borderline outcomes (incidence of vomiting, patient-reported perception of postoperative ileus) reached consensus to be added according to the predefined threshold for consensus (*Table 1*).

During the meeting, the agreed outcomes ‘incidence of postoperative ileus’ and ‘incidence of prolonged postoperative ileus’ were considered to be markedly similar, and consensus was reached to combine these into a single construct ‘incidence of postoperative ileus’ (12 of 13 agreed, 2 abstentions). It was also noted that four agreed outcomes (abdominal infection, anastomotic leak, peritonitis, enterotomy) were akin to risk factors rather than conventional outcomes of ileus. Consensus was reached to retain these to reflect essential contextual information required alongside other outcomes in the set (12 of 12 agreed, 3 abstentions).

A final core outcome set comprising 24 outcomes was agreed (*Table 3*). Consensus was achieved to group outcomes into domains to reflect the patient journey and to rationalize the presentation of the set. Clustering of outcomes was achieved wholly through consensus, with the final wording of domains finalized by the steering committee. This produced a total of six domains along with three outcomes that remained ungrouped.

**Table 3:** Final core outcome set for postoperative ileus

Domain	Core outcome
**Incidence and duration of ileus**	Incidence of ileus
	Duration of ileus
**Vomiting and gastric decompression**	Incidence of nausea
	Incidence of vomiting
	Duration of vomiting
	Need for nasogastric tube placement
	Volume of nasogastric tube aspirate
**Abdominal pain**	Severity of abdominal pain
**Nutritional factors**	Nutritional status
	Time without adequate nutritional intake
	Need for parenteral nutrition
**Return of gut function**	A measure of gastrointestinal recovery using a validated tool
	Time to first stoma output
	Readiness for discharge based on gastrointestinal function
**Patient experience**	Patient-reported perception of ileus
**Complications arising from ileus**	Morbidity
	Septic complications
	Admission to intensive care
	Organ injury or failure
**Readmission**	Readmission
**Predisposing factors for ileus**	Abdominal infection
	Anastomotic leak
	Peritonitis
	Enterotomy

## Discussion

An agreed core outcome set for postoperative ileus after intestinal surgery is presented. This was developed through a rigorous process with input from key stakeholder groups and with focus on the patient voice. This should now provide a universal framework for evaluating the effectiveness of clinical interventions to reduce ileus after intestinal surgery. Importantly, all agreed outcomes within the set are essential, but this does not restrict the use of other outcomes available to investigators. Instead, it provides a minimum standard to normalize outcome selection and to improve comparability when implementing research into practice.

A strength of this study is that all decisions were made through multidisciplinary consensus. This ensured that challenging points of contention were addressed openly and with the collective involvement of all stakeholders. Another strength is the international scope of recruitment across continents. This will ensure that the final set is applicable across broad settings and therefore more likely to be adopted universally. Limitations are also recognized. It is acknowledged that recruitment to the study favoured participants with access to the internet and English as a first language, which may have implications for its generalizability. It is also acknowledged that bias may have occurred during the consensus meeting in favour of opinions that were expressed most assertively. This was mitigated as far as possible by an experienced independent chairperson along with support from patient representatives.

The development of this core outcome set is the first step towards standardizing outcome selection and reporting in studies of postoperative ileus. The next stage is to define a series of outcome instruments within an agreed core measurement set. This will be particularly important for the core outcome ‘incidence and duration of ileus’, which is contingent on an accepted definition for ileus. Although this is beyond the scope of the present study, it is notable that ‘interval from surgery until passage of flatus/stool *and* tolerance of an oral diet’ has been proposed elsewhere as a suitable definition through previous expert consensus^13^.

A core outcome set for clinical studies of postoperative ileus after intestinal surgery has been developed. This provides a standard framework to evaluate the effectiveness of clinical interventions. Its adoption is encouraged to increase the value of future research related to post-operative ileus after intestinal surgery, and to facilitate informed decision-making when implementing changes in clinical practice.

## Collaborators

S.J. Chapman (University of Leeds, Leeds, UK), M.J. Lee (University of Sheffield, Sheffield, UK), S. Blackwell (Public Representative), R. Arnott (Green Templeton College, Oxford, UK), R.P.G. ten Broek (Radboud University Medical Center, Nijmegen, Netherlands), C.P. Delaney (Cleveland Clinic, Cleveland, USA), N.N. Dudi-Venkata (University of Adelaide, Adelaide, Australia), R. Fish (University of Manchester, Manchester, UK), D. Hind (University of Sheffield, Sheffield, UK), D.G. Jayne (University of Leeds, Leeds, UK), K. Mellor (University of Oxford, Oxford, UK), A. Mishra (Maulana Azad Medical College, New Delhi, India), G. O’Grady, (University of Auckland, Auckland, New Zealand), T. Sammour (Royal Adelaide Hospital, Adelaide, Australia), G. Thorpe (University of East Anglia, Norwich, UK), C.I. Wells (University of Auckland, Auckland, New Zealand), A.M. Wolthuis (University Hospital Leuven, Leuven, Belgium), N.S. Fearnhead (Cambridge University Hospitals NHS Foundation Trust, Cambridge, UK), S. Adegbola, G. Bagaglini, M. Bath, N. Bibby, C. Bisset, N. Blefari, N.S. Blencowe, W. Bolton, J.P. Bulte, J. Burch, M. Campanelli, O. Cano-Valderrama, J. Carver, C. Challand, S. Chan, S. Chandler, D. Clerc, P. Coe, D. Cox, K.L.R. Cross, A. Culkin, V. Cuthill, S. Daniels, A. Dawson, L. Dawson, F. Dixon, C. Downey, T. Drake, S. Duff, G. Dunning, E. Espin-Basany, M.D. Evans, M. Fakhrul-Aldeen, N. Fisher, S. Fleetwood-Beresford, G. Gallo, Z. Garoufalia, R. George, J. Han, D. Harji, R. Harmston, D.A. Harris, M. Mohammed, J. Helliwell, J. Hepburn, P. Herrod, N. Horwood, C. Keane, S. Kelly, H.M. Kroon, M.D.S. Lonsdale, G. Major, J. Mattison, A. Lawson McLean, M. Millan, S. Limbert, F. McDermott, A. Mehraj, C. Moriarty, S. Moug, E. Murray, M. Naylor, D. Nepogodiev, J. Oliver, D. Pandey, F. Pata, H.M. Paterson, A. Peckham-Cooper, G. Pellino, P. Pockney, V.K. Proctor, D. Proud, V. Rew, M. Rutegård, K. Sahnan, A. Sayers, L. Siragusa, R.W. Smillie, J. Spratt, D. Swain, S. Taylor, P. Tejedor, O. Thomas, J. Thompson, K. Tsimogiannis, D. Tuohey, R. Vissapragada, M.U. Younis, P.G. Vaughan-Shaw, K. Whyte, K. Wheelband, A. Williams, A. Yates, R. Young.

## Supplementary Material

znac052_Supplementary_Data
