# Supplementary material for: Core outcome set for clinical studies of postoperative ileus after intestinal surgery
Source: Br J Surg. 2022 Mar 8;109(6):493–6. doi: 10.1093/bjs/znac052 (PMC10364764; doi:10.1093/bjs/znac052)
Supplement: znac052_Supplementary_Data [file znac052_supplementary_data.docx]

**Supplementary Material**

*The following supplementary material should be read in conjunction with the abridged report in the main text.*

1. Appendix S1 - Supplementary Methods & Results
2. Appendix S2 - Authorship and collaborators
3. Figure S1 – Summary of outcome longlisting
4. Box S1 – Additional outcomes added after Round 1
5. Table S1 – Plain English summaries of all outcomes
6. Table S2 – Results of the Delphi Process
7. Table S3 – Data for attrition bias and consensus meeting sampling bias

**Appendix S1 - Supplementary Methods & Results**

***Supplementary Methods***

*Ethics & Governance*

The study was registered with the COMET Initiative prior to the start of the study (<https://cometinitiative.org/Studies/Details/1479>). Across the abridged summary of the study and the present Supplementary Methods, the findings are reported in line with the Core Outcome Set - Standards for Reporting (COS-STAR) guidelines.^S1^

*Protocol Variations*

In 2020, coronavirus disease 2019 (COVID-19) was declared as a global pandemic by the World Health Organisation. Social distancing regulations enforced by national legislatures precluded the planned face-to-face consensus meeting due to take place in Auckland, New Zealand in November 2020. Following consultation within the Steering Committee, a decision was made to convert the consensus meeting to a virtual event via an online teleconference service. Care was taken to ensure inclusive and fair participation throughout by means of an event facilitator (SJC) and an independent chairperson (RF). A substantial amendment to the approved protocol was confirmed on 11^th^ January 2021 by the University of Sheffield Research Ethics Committee prior to the online consensus event (6^th^ March 2021).

In contrast to the published protocol (first online 30^th^ November 2019), but prior to the start of data collection (17^th^ January 2020), the Delphi process criteria for consensus were amended. This was done following careful consideration between members of the Steering Committee and with close involvement of patient representatives. Previous criteria for consensus were defined as ≥70% of participants in each stakeholder group assigning a score of 7-9 or ≤15% of participants in each group indicating a score of 1-3. The latter component of these criteria was considered to be insufficiently discriminative and was removed. An additional criterion for consensus (≥90% in a single key stakeholder group) was set to recognise outcomes that were of strong importance to individual groups and in particular to patients.

*Study Overview*

The study consisted of three phases according to Delphi methodology and was conducted between 17^th^ January 2020 and 6th March 2021.^S2^ In Phase 1, a longlist of candidate outcomes was generated from a systematic review, international stakeholder focus groups, and consultation within the steering committee. In Phase 2, candidate outcomes were presented to stakeholders, followed by voting on their importance using numerical rating scales. In Phase 3, an online, consensus meeting, with representation from key stakeholder groups, was convened to ratify the final outcome set and finalise it for dissemination.

*Information Sources*

Firstly, outcomes reported in existing research were identified from a systematic review of published literature that has been previously reported.^S3^ In brief, outcomes relating to the recovery of intestinal function were extracted from reports of randomised controlled trials evaluating any intervention to reduce ileus after intestinal surgery between 1990 and 2017 (PROSPERO: CRD42017082351). Secondly, outcomes of perceived importance amongst stakeholders were identified from international focus groups using a Nominal Group Technique approach.^S4^ Four groups were convened, including one patient group (n=3 participants), one allied healthcare professional group (n=3 participants), and two medical professional groups (n=5 and n=3 participants). During these groups, additional unique outcomes were proposed, discussed within the groups, and agreed to be taken forward or discarded. Finally, all outcomes identified from the systematic review and focus groups were combined to generate a draft longlist. This was reviewed and ratified by the steering committee, with composite outcomes broken down to produce discrete constructs, duplicated items consolidated to avoid recurring repetition, and a small number of additional outcomes added where it was felt that this improved international applicability. Plain English summaries were produced by patient representatives for all outcomes included in the final longlist and agreed with the wider Steering Committee (Supplementary Table 1).

*Delphi Process*

An online, three-round Delphi process was facilitated using the Google Forms platform (Google, Mountain View, CA) to prioritise longlisted outcomes. During each round, outcomes and plain English summaries were presented to participants in a random order alongside a nine-point numerical rating scale, as recommended by the Grading of Recommendations Assessment, Development and Evaluation (GRADE) working group.^S5^ Higher scores indicated an outcome of greater importance. During Round 1, participants were asked to rate all longlisted outcomes. Those that fulfilled a pre-defined threshold of consensus were carried forward to the consensus meeting (see Box 1 for consensus thresholds). Suggestions for additional outcomes were invited during Round 1 and these were considered by the steering committee for scope and subsequent inclusion. During Rounds 2 and 3, participants were shown a summary of how they and other stakeholder groups had rated outcomes in each of the earlier rounds. This was presented numerically as individual participant scores alongside median scores for all stakeholder groups. All remaining and additional outcomes that had not achieved consensus were then summarised, re-presented to participants, and carried forward to the consensus meeting or discarded according to the same criteria.

*Consensus Meeting*

The results of the Delphi process were discussed during an online consensus meeting. Participants were eligible to participate if they had completed all three rounds of the Delphi process and were purposively sampled to represent all key stakeholder groups across a diverse international setting. The meeting was chaired by an independent chairperson with specific expertise in the development of core outcome sets and COMET methodology (RF).

After careful consideration by the steering committee and advocacy from patient representatives, it was decided to re-present borderline outcomes that had narrowly missed the criteria for consensus during the Delphi process for further detailed discussion. To facilitate this, an extended threshold for consensus was applied (Main Text, Box 1). It was also decided to re-present one outcome - “time to tolerate fluid intake” - due to erratic and inconsistent scoring pattens observed between Rounds 2 and 3, which the committee felt may have indicated some underlying uncertainty in voting responses. All borderline outcomes were discussed verbally in real time alongside webchat functions and diverging views were actively sought. A final, anonymised vote took place on whether to include these outcomes in the final outcome set using the same threshold for consensus as in previous rounds.

The remainder of the consensus meeting focussed on ratification, wording, and presentation of outcomes that had achieved consensus during the earlier Delphi process. Proposed wording and suggestions for presentation were generated through discussion and presented to participants as discrete statements for voting. Dichotomous voting took place and decisions were ratified according to a meeting-specific threshold of consensus (Main text, Box 1).

*Additional Analyses*

All analyses were presented descriptively as medians and interquartile ranges. The presence of attrition bias was explored by comparing Round 1 summary scores (medians) of participants who did and did not subsequently take part in Round 2. The presence of sampling bias during the consensus meeting was explored by comparing Round 2 summary scores (medians) of participants who did and did not take part in the consensus meeting.

***Supplementary Results***

*Consensus Meeting*

As reported in the main text, the borderline outcome “gastrointestinal-related quality of life” reached consensus for inclusion after consideration during the consensus meeting. Of note, attendees felt that the outcome should be re-phrased for greater clarity. The main reason for this was because “quality of life” was considered to reflect longer-term implications on health and well-being which were not within remit of the present core outcome set. Consensus was achieved to re-phrase this outcome to “patient-reported perception of post-operative ileus” (n=12/14; in agreement; 1 abstention) and is referred to as such in the main text.

*Additional Analyses*

An attrition rate of 20.6% was observed between Round 1 (n=155 responses) and Round 2 (n=123) of the Delphi process. This was greatest amongst allied healthcare professionals (33.3%), followed by patients (19.5%), and medical professionals (18.3%). The median of differences in per-outcome scores across Rounds 1 and 2 was 0 (IQR: 0-1). Sampling bias within the consensus meeting was explored by comparing Round 2 scores of participants who took part in the meeting with scores of those who were not sampled. The median difference was 0 (IQR: 0-1). A full outline of data is summarised in Supplementary Table 3.

**References**

S1. Kirkham JJ. Gorst S. Altman DG. et al. Core Outcome Set – STAndards for Reporting: The COS-STAR Statement. *Plos Med* 2016;13:e1002148.

S2. Keeley T. Williamson P. Callery P. et al. The use of qualitative methods to inform Delphi surveys in core outcome set development. *Trials* 2016;17:230.

S3. Chapman SJ. Thorpe G. Vallance AE. et al. Association of Coloproctology of Great Britain and Ireland Gastrointestinal Recovery Group. Systematic review of definitions and outcome measures for return of bowel function after gastrointestinal surgery. *BJS Open* 2018;3:1-10.

S4. Horton JN. Nominal group technique. A method of decision-making by committee. *Anaesthesia* 1980;35:811-814.

S5. The Grading of Recommendations Assessment, Development and Evaluation (short GRADE) working group. Available at: <https://www.gradeworkinggroup.org> [Accessed 4^th^ August 2021].

**Appendix S2 - Authorship and collaborators**

**Writing Group (authors)**

SJ Chapman*, MJ Lee, S Blackwell, R Arnott, RPG ten Broek, CP Delaney, NN Dudi-Venkata, R Fish, D Hind, DG Jayne, K Mellor, A Mishra, G O’Grady, T Sammour, G Thorpe, CI Wells, AM Wolthuis, NS Fearnhead *(senior author and guarantor)*

**Indicates first authorship*

**Study Collaborators**

S Adegbola, G Bagaglini, M Bath, N Bibby, C Bisset, N Blefari, NS Blencowe, W Bolton, JP Bulte, J Burch, M Campanelli, O Cano-Valderrama, J Carver, C Challand, S Chan, S Chandler, D Clerc, P Coe, D Cox, KLR Cross, A Culkin, V Cuthill, S Daniels, A Dawson, L Dawson, F Dixon, C Downey, T Drake, S Duff, G Dunning, E Espin-Basany, MD Evans, M Fakhrul‐Aldeen, N Fisher, S Fleetwood-Beresford, G Gallo, Z Garoufalia, R George, J Han, D Harji, R Harmston, DA Harris, M Mohammed, J Helliwell, J Hepburn, P Herrod, N Horwood, C Keane, S Kelly, HM Kroon, MDS Lonsdale, G Major, J Mattison, A Lawson McLean, M Millan, S Limbert, F McDermott, A Mehraj, C Moriarty, S Moug, E Murray, M Naylor, D Nepogodiev, J Oliver, D Pandey, F Pata, HM Paterson, A Peckham-Cooper, G Pellino, P Pockney, VK Proctor, D Proud, V Rew, M Rutegård, K Sahnan, A Sayers, L Siragusa, RW Smillie, J Spratt, D Swain, S Taylor, P Tejedor, Thomas O, J Thompson, K Tsimogiannis, D Tuohey, R Vissapragada, MU Younis, PG Vaughan-Shaw, C Wells, K Whyte, K Wheelband, A Williams, A Yates, R Young

**Box S1: Additional outcomes added after Round 1**

**Suggested by medical professionals:**

- Length of hospital stay
- Time without adequate nutritional intake
- Incidence of hypokalaemia
- Cost of admission

**Suggested by patients:**

- Need for intensive care unit admission
- Mental well-being
- Weight loss
- Need for readmission (for any reason)

**Figure S1 – Summary of outcome longlisting**

**
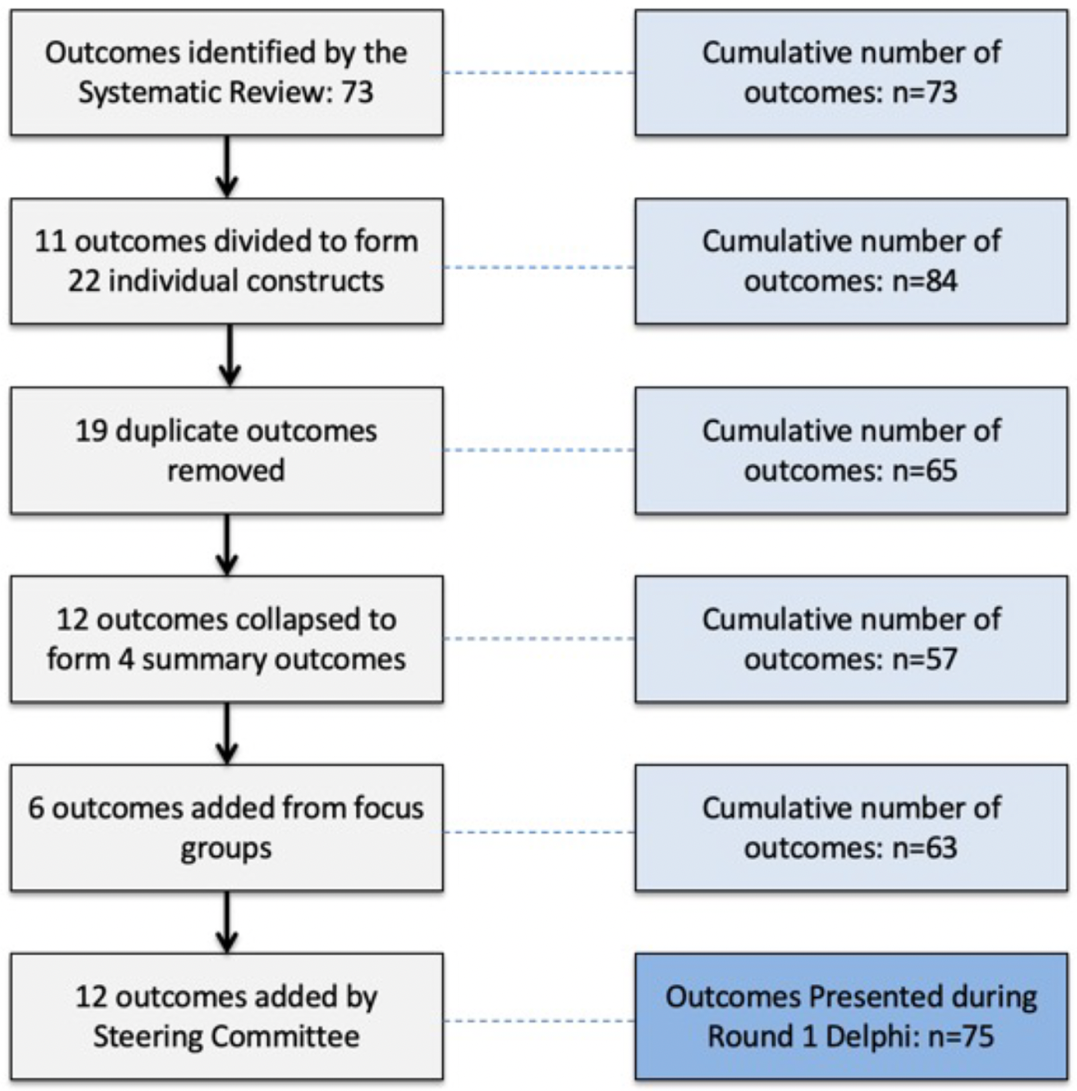
**

**Table S1 - Plain English summaries for all outcomes**

| **Outcomes identified prior to Delphi Process** | | |
| --- | --- | --- |
| **No.** | **Outcome** | **Plain English Summary** |
| 1 | Time to first fluid intake | How long it takes after the operation to have a drink |
| 2 | Time to tolerate fluid intake | How long it takes after the operation to have a drink and not feel sick afterwards |
| 3 | Time to intake of > 1000 ml fluids per day | How long it takes after the operation to be able to drink more than 1 litre in a day |
| 4 | Time to first soft food | How long it takes after the operation to eat soft food |
| 5 | Time to tolerate low-residue diet | How long it takes after the operation to eat low fibre food and not feel sick afterwards |
| 6 | Time to first solid intake | How long it takes after the operation to eat solid food |
| 7 | Time to tolerate solid intake | How long it takes after the operation to eat solid food and not feel sick afterwards |
| 8 | Time to tolerate normal diet | How long it takes after the operation to eat a normal diet and not feel sick afterwards |
| 9 | Amount of food intake per meal | How much you are able to eat at mealtimes |
| 10 | Incidence of nausea | If you feel sick after the operation |
| 11 | Duration of nausea | How long you feel sick for after the operation |
| 12 | Incidence of vomiting | If you are being sick after the operation |
| 13 | Duration of vomiting | How long you are being sick for after the operation |
| 14 | Incidence of abdominal pain | If you have stomach pain after the operation |
| 15 | Severity of abdominal pain | Severity of stomach pain after the operation |
| 16 | Incidence of abdominal swelling/distension | If your stomach is swollen after the operation |
| 17 | Severity of abdominal swelling/distension | Severity of stomach swelling after the operation |
| 18 | Need for laxative medication | Whether you need laxative medication to help you poo |
| 19 | Need for antiemetic medication | Whether you need medication to stop you feeling/being sick |
| 20 | Need for parenteral nutrition | If you need liquid food via a drip/feeding line after the operation |
| 21 | Duration of parenteral nutrition | How long you need to be given liquid food via a drip/feeding line |
| 22 | Time to first passage of flatus | How long it takes after your operation to pass wind |
| 23 | Time to second passage of flatus | How long it takes after your operation to pass wind for the second time |
| 24 | Cumulative frequency of flatus | How often you pass wind after the operation |
| 25 | Time to first passage of stool | How long it takes after your operation to have a poo |
| 26 | Frequency of stool | How often after your operation you need to go for a poo (whilst still in hospital) |
| 28 | Consistency of stool | How hard or soft your poo is after the operation |
| 28 | Time to return of appetite | How long it takes after the operation to feel like you want to eat |
| 29 | Extent of hunger | How hungry you feel after the operation |
| 30 | Incidence of satiety | If you feel full after eating |
| 31 | Extent of satiety | How full you feel after eating |
| 32 | Incidence of belching | If you are belching after the operation |
| 33 | Duration of belching | How long after the operation that you are belching |
| 34 | Incidence of hiccups | If you have hiccups after the operation |
| 35 | Gastrointestinal-related quality of life | How your bowel function is impacting on your quality of life (whilst in hospital) |
| 36 | Time to first stoma output | If you have a stoma, how long after your operation it takes for it to work |
| 37 | Incidence of postoperative ileus | If your bowel "goes to sleep" after your operation |
| 38 | Incidence of prolonged postoperative ileus | If your bowel "goes to sleep" for longer than expected after your operation |
| 39 | Duration of postoperative ileus | How long your bowel is "asleep" after your operation |
| 40 | Time to GI Recovery using a validated tool | How long your bowel takes to recover its function |
| 41 | Need for nasogastric tube placement | If you need a tube inserted up your nose to help your bowel recover |
| 42 | Duration of nasogastric tube placement | How long you need a tube inserted up your nose to help your bowel to recover |
| 43 | Volume of nasogastric tube aspirate | If you have a tube inserted up your nose, how much fluid is coming out of it |
| 44 | Incidence of nasogastric tube aspirate > 500 ml per day | If you have a tube up your nose, is it draining more than 500mls of fluid a day |
| 45 | Vomiting after nasogastric tube removal | If you are sick after having the tube up your nose removed |
| 46 | Time to first postoperative abdominal peristalsis | How long it takes after your operation for your bowel to physically move/churn |
| 47 | Time to detect bowel sounds | How long it takes after your operation to hear noises from your bowel |
| 48 | Frequency of bowel sounds | How often your bowel rumbles/makes noises |
| 49 | Incidence of readmission due to postoperative ileus | If you have to return to hospital due to your bowel still not working properly |
| 50 | Readiness for discharge based on gastrointestinal function | If you are ready to go home based on how well your bowel is working |
| 51 | Incidence of morbidity due to postoperative ileus | If you become ill after your operation as a result of your bowels not working |
| 52 | Nutritional status | How well-nourished your body is |
| 53 | Gastrointestinal motility | How much your bowel physically moves/churns |
| 54 | Gastric emptying | How quickly food/fluids empty into the bowel from the stomach |
| 55 | Gastrointestinal transit | How quickly food/fluids move from the start of the gut to the end |
| 56 | Quantification of bowel gas | How much gas is in your bowel |
| 57 | Complications: Urinary | Problems with the bladder after the operation |
| 58 | Complications: Respiratory | Problems with the lungs after the operation |
| 59 | Complications: Pneumonia | A chest infection after the operation |
| 60 | Complications: Organ injury or failure | Organ failure after the operation |
| 61 | Complications: Thrombosis or embolism | Blood clots in the legs or lungs after the operation |
| 62 | Complications: Renal | Problems with the kidneys after the operation |
| 63 | Complications: Sepsis | Severe infection (septicaemia) after the operation |
| 64 | Complications: Cardiac | Problems with the heart after the operation |
| 65 | Complications: Abdominal infection | Infection inside the belly after the operation |
| 66 | Complications: Peritonitis | Inflammation inside the belly after the operation |
| 67 | Complications: Enterotomy | A hole in the bowel made accidentally during the operation |
| 68 | Complications: Anastomotic leak | A leak in the join between two pieces of bowel |
| 69 | Complications: Wound infection | An infection of the wound after the operation |
| 70 | Anxiety | How nervous you feel after the operation |
| 71 | Vomiting with Nasogastric tube in situ | If you are being sick whilst the tube up your nose is in place |
| 72 | Postoperative inflammatory response | How inflamed your bowel and other tissues are after the operation |
| 73 | Radiological intestinal dilatation | How swollen your intestines look on a scan |
| 74 | Overall fluid balance | How hydrated (or dehydrated) you are after the operation |
| 75 | Mobility | How much you can move around after the operation |
| **Additional Outcomes Added during Round 1 of the Delphi Survey** | | |
| 76 | Length of hospital stay | How long you stay in hospital |
| 77 | Need for intensive care unit admission | If you need to go to intensive care |
| 78 | Mental well-being | How well you feel in yourself/mental health |
| 79 | Weight loss | How much weight you have lost |
| 80 | Time without adequate nutritional intake | Amount of time without adequate food intake |
| 81 | Need for readmission (for any reason) | If you are re-admitted to hospital for any reason |
| 82 | Incidence of hypokalaemia | If you develop low potassium levels (a salt found in the blood) |
| 83 | Cost of admission | Healthcare costs (to the health system) |

**Table S2 – Results of the Delphi Process**

| **Outcome** | **Medical Professionals** | | | **Allied Healthcare Professionals** | | | **Patients** | | |
| --- | --- | --- | --- | --- | --- | --- | --- | --- | --- |
|  | **R1 (7-9)**  **(n=93)** | **R2 (7-9)**  **(n=76)** | **R3 (7-9)**  **(n=71)** | **R1 (7-9)**  **(n=21)** | **R2 (7-9)**  **(n=14)** | **R3 (7-9)**  **(n=12)** | **R1 (7-9)**  **(n=41)** | **R2 (7-9)**  **(n=33)** | **R3 (7-9)**  **(n=29)** |
| ***Outcomes taken forward to the consensus meeting*** | | | | | | | | | |
| Incidence of readmission due to postoperative ileus | 88.17% | NR | NR | 90.48% | NR | NR | 87.80% | NR | NR |
| A measure of gastrointestinal recovery using a validated tool | 78.49% | NR | NR | 76.19% | NR | NR | 82.93% | NR | NR |
| Need for parenteral nutrition | 81.72% | NR | NR | 95.24% | NR | NR | 63.41% | NR | NR |
| Time to first stoma output | 78.49% | NR | NR | 90.48% | NR | NR | 75.61% | NR | NR |
| Need for nasogastric tube placement | 84.95% | NR | NR | 90.48% | NR | NR | 70.73% | NR | NR |
| Severity of abdominal pain | 40.86% | NR | NR | 57.14% | NR | NR | 92.68% | NR | NR |
| Incidence of postoperative ileus | 87.10% | NR | NR | 90.48% | NR | NR | 85.37% | NR | NR |
| Duration of postoperative ileus | 86.02% | NR | NR | 95.24% | NR | NR | 82.93% | NR | NR |
| Incidence of prolonged postoperative ileus | 92.47% | NR | NR | 95.24% | NR | NR | 85.37% | NR | NR |
| Incidence of morbidity due to postoperative ileus | 82.80% | NR | NR | 100.00% | NR | NR | 87.80% | NR | NR |
| Complications: Anastomotic leak | 83.87% | NR | NR | 85.71% | NR | NR | 90.24% | NR | NR |
| Readiness for discharge based on gastrointestinal function | 70.97% | NR | NR | 76.19% | NR | NR | 80.49% | NR | NR |
| Complications: Enterotomy | 58.06% | NR | NR | 66.67% | NR | NR | 90.24% | NR | NR |
| Nutritional status | 67.74% | 73.68% | NR | 71.43% | 92.86% | NR | 65.85% | 63.64% | NR |
| Volume of nasogastric tube aspirate | 53.76% | 64.47% | NR | 85.71% | 92.86% | NR | 53.66% | 75.76% | NR |
| Duration of vomiting | 63.44% | 64.47% | NR | 71.43% | 92.86% | NR | 70.73% | 72.73% | NR |
| Complications: Abdominal infection | 60.22% | 69.74% | NR | 57.14% | 64.29% | NR | 82.93% | 93.94% | NR |
| Incidence of nausea | 53.76% | 56.58% | NR | 76.19% | 92.86% | NR | 63.41% | 54.55% | NR |
| Complications: Peritonitis | 65.59% | 76.32% | NR | 66.67% | 71.43% | NR | 70.73% | 72.73% | NR |
| Complications: Sepsis | 62.37% | 72.37% | NR | 66.67% | 71.43% | NR | 87.80% | 90.91% | NR |
| Need for intensive care unit admission* | NR | 63.16% | NR | NR | 71.43% | NR | NR | 96.97% | NR |
| Time without adequate nutritional intake* | NR | 71.05% | NR | NR | 85.71% | NR | NR | 75.76% | NR |
| Complications: Organ injury or failure | 67.74% | 67.11% | 70.42% | 66.67% | 71.43% | 58.33% | 80.49% | 87.88% | 93.10% |
| ***Borderline outcomes taken forward to the consensus meeting*** | | | | | | | | | |
| Incidence of vomiting | 74.19% | 72.37% | 76.06% | 71.43% | 78.57% | 66.67% | 68.29% | 66.67% | 65.52% |
| Incidence of nasogastric tube aspirate > 500 ml per day | 58.06% | 65.79% | 67.61% | 71.43% | 78.57% | 66.67% | 58.54% | 72.73% | 65.52% |
| Time to tolerate normal diet | 69.89% | 64.47% | 66.20% | 71.43% | 71.43% | 75.00% | 48.78% | 57.58% | 65.52% |
| Time to first passage of stool | 64.52% | 64.47% | 64.79% | 85.71% | 71.43% | 83.33% | 73.17% | 75.76% | 68.97% |
| Gastrointestinal-related quality of life | 63.44% | 69.74% | 69.01% | 57.14% | 64.29% | 75.00% | 78.05% | 69.70% | 72.41% |
| Time to tolerate fluid intake | 72.04% | 67.11% | 60.56% | 71.43% | 85.71% | 75.00% | 68.29% | 69.70% | 62.07% |
| ***Outcomes not reaching consensus (not taken forward to the consensus meeting)*** | | | | | | | | | |
| Frequency of bowel sounds | 15.05% | 13.16% | 11.27% | 61.90% | 42.86% | 33.33% | 43.90% | 39.39% | 34.48% |
| Complications: Renal | 45.16% | 44.74% | 38.03% | 42.86% | 42.86% | 25.00% | 73.17% | 69.70% | 72.41% |
| Gastric emptying | 31.18% | 28.95% | 16.90% | 61.90% | 57.14% | 41.67% | 60.98% | 45.45% | 27.59% |
| Mobility | 60.22% | 55.26% | 60.56% | 52.38% | 64.29% | 50.00% | 68.29% | 66.67% | 68.97% |
| Incidence of abdominal pain | 45.16% | 42.11% | 47.89% | 52.38% | 50.00% | 50.00% | 85.37% | 69.70% | 62.07% |
| Radiological intestinal dilatation | 31.18% | 26.32% | 30.99% | 71.43% | 64.29% | 58.33% | 58.54% | 63.64% | 31.03% |
| Anxiety | 23.66% | 17.11% | 14.08% | 52.38% | 42.86% | 25.00% | 58.54% | 45.45% | 41.38% |
| Need for laxative medication | 27.96% | 19.74% | 9.86% | 52.38% | 57.14% | 41.67% | 41.46% | 45.45% | 24.14% |
| Quantification of bowel gas | 17.20% | 9.21% | 12.68% | 47.62% | 42.86% | 25.00% | 43.90% | 33.33% | 31.03% |
| Time to first postoperative abdominal peristalsis | 25.81% | 26.32% | 29.58% | 57.14% | 50.00% | 50.00% | 63.41% | 66.67% | 58.62% |
| Incidence of satiety | 20.43% | 15.79% | 8.45% | 42.86% | 42.86% | 8.33% | 29.27% | 30.30% | 10.34% |
| Complications: Urinary | 22.58% | 27.63% | 18.31% | 42.86% | 28.57% | 25.00% | 75.61% | 75.76% | 62.07% |
| Complications: Pneumonia | 58.06% | 56.58% | 60.56% | 38.10% | 50.00% | 41.67% | 63.41% | 78.79% | 75.86% |
| Vomiting after nasogastric tube removal | 68.82% | 59.21% | 59.15% | 71.43% | 78.57% | 75.00% | 53.66% | 66.67% | 62.07% |
| Time to first solid intake | 60.22% | 55.26% | 53.52% | 76.19% | 85.71% | 33.33% | 58.54% | 39.39% | 58.62% |
| Time to return of appetite | 47.31% | 34.21% | 21.13% | 61.90% | 71.43% | 50.00% | 41.46% | 39.39% | 37.93% |
| Overall fluid balance | 54.84% | 59.21% | 50.70% | 76.19% | 78.57% | 75.00% | 70.73% | 69.70% | 65.52% |
| Postoperative inflammatory response | 50.54% | 47.37% | 39.44% | 66.67% | 35.71% | 41.67% | 73.17% | 72.73% | 65.52% |
| Duration of parenteral nutrition | 75.27% | 78.95% | 80.28% | 80.95% | 78.57% | 66.67% | 63.41% | 66.67% | 58.62% |
| Time to first passage of flatus | 70.97% | 60.53% | 64.79% | 80.95% | 64.29% | 66.67% | 53.66% | 63.64% | 51.72% |
| Extent of satiety | 21.51% | 17.11% | 9.86% | 47.62% | 35.71% | 8.33% | 41.46% | 30.30% | 17.24% |
| Duration of belching | 20.43% | 14.47% | 11.27% | 47.62% | 50.00% | 16.67% | 29.27% | 15.15% | 13.79% |
| Amount of food intake per meal | 26.88% | 25.00% | 18.31% | 52.38% | 42.86% | 16.67% | 36.59% | 30.30% | 24.14% |
| Time to second passage of flatus | 30.11% | 19.74% | 16.90% | 66.67% | 71.43% | 33.33% | 46.34% | 33.33% | 27.59% |
| Cumulative frequency of flatus | 34.41% | 23.68% | 16.90% | 66.67% | 42.86% | 41.67% | 39.02% | 45.45% | 20.69% |
| Complications: Thrombosis or embolism | 43.01% | 40.79% | 39.44% | 38.10% | 28.57% | 25.00% | 75.61% | 84.85% | 75.86% |
| Time to detect bowel sounds | 21.51% | 13.16% | 16.90% | 61.90% | 35.71% | 16.67% | 51.22% | 42.42% | 37.93% |
| Need for antiemetic medication | 41.94% | 35.53% | 35.21% | 61.90% | 50.00% | 41.67% | 56.10% | 57.58% | 44.83% |
| Complications: Cardiac | 41.94% | 40.79% | 40.85% | 38.10% | 28.57% | 25.00% | 70.73% | 78.79% | 86.21% |
| Duration of nasogastric tube placement | 67.74% | 65.79% | 69.01% | 80.95% | 85.71% | 58.33% | 65.85% | 75.76% | 75.86% |
| Consistency of stool | 17.20% | 11.84% | 12.68% | 47.62% | 42.86% | 8.33% | 46.34% | 39.39% | 34.48% |
| Extent of hunger | 30.11% | 22.37% | 18.31% | 33.33% | 64.29% | 50.00% | 26.83% | 24.24% | 24.14% |
| Time to first soft food | 40.86% | 34.21% | 36.62% | 71.43% | 57.14% | 50.00% | 46.34% | 57.58% | 55.17% |
| Frequency of stool | 23.66% | 25.00% | 21.13% | 57.14% | 57.14% | 58.33% | 60.98% | 63.64% | 62.07% |
| Vomiting with nasogastric tube in situ | 48.39% | 53.95% | 54.93% | 61.90% | 85.71% | 83.33% | 75.61% | 78.79% | 72.41% |
| Incidence of abdominal swelling/distension | 40.86% | 40.79% | 26.76% | 71.43% | 78.57% | 66.67% | 56.10% | 48.48% | 48.28% |
| Duration of nausea | 48.39% | 42.11% | 43.66% | 66.67% | 64.29% | 50.00% | 73.17% | 60.61% | 48.28% |
| Complications: Respiratory | 51.61% | 55.26% | 57.75% | 42.86% | 50.00% | 41.67 | 70.73% | 81.82% | 72.41% |
| Complications: Wound infection | 60.22% | 40.79% | 42.25% | 42.86% | 42.86% | 41.67% | 82.93% | 78.79% | 82.76% |
| Incidence of hiccups | 38.71% | 30.26% | 22.54% | 52.38% | 57.14% | 41.67% | 24.39% | 27.27% | 10.34% |
| Severity of abdominal swelling/distension | 43.01% | 39.47% | 38.03% | 76.19% | 85.71% | 50.00% | 65.85% | 66.67% | 68.97% |
| Time to tolerate solid intake | 65.59% | 55.26% | 66.20% | 66.67% | 78.57% | 75.00% | 51.22% | 66.67% | 58.62% |
| Time to tolerate low-residue diet | 47.31% | 34.21% | 28.17% | 57.14% | 71.43% | 41.67% | 56.10% | 54.55% | 37.93% |
| Incidence of belching | 25.81% | 23.68% | 18.31% | 42.86% | 50.00% | 41.67% | 31.71% | 24.24% | 13.79% |
| Gastrointestinal motility | 35.48% | 28.95% | 29.58% | 61.90% | 50.00% | 41.67% | 51.22% | 57.58% | 44.83% |
| Time to intake of > 1000 ml fluids per day | 41.94% | 38.16% | 45.07% | 57.14% | 42.86% | 41.67% | 60.98% | 51.52% | 48.28% |
| Gastrointestinal transit | 37.63% | 39.47% | 32.39% | 61.90% | 57.14% | 41.67% | 56.10% | 54.55% | 44.83% |
| Time to first fluid intake | 50.54% | 55.26% | 46.48% | 71.43% | 64.29% | 58.33% | 65.85% | 51.52% | 55.17% |
| Length of hospital stay* | NR | 71.05% | 66.20% | NR | 64.29% | 41.67% | NR | 63.64% | 55.17% |
| Mental well-being* | NR | 38.16% | 45.07% | NR | 64.29% | 66.67% | NR | 69.70% | 72.41% |
| Weight loss* | NR | 47.37% | 38.03% | NR | 50.00% | 33.33% | NR | 39.39% | 41.38% |
| Incidence of hypokalaemia* | NR | 46.05% | 38.03% | NR | 57.14% | 41.67% | NR | 48.48% | 44.83% |
| Cost of admission* | NR | 55.26% | 39.44% | NR | 35.71% | 41.67% | NR | 27.27% | 20.69% |
| Need for readmission (for any reason)* | NR | 71.05% | 69.01% | NR | 64.29% | 50.00% | NR | 84.85% | 89.66% |

*As per the Method, participants were invited to propose new outcomes at the end of Round 1. Proposals considered to be within scope by the Steering Committee and subsequently presented to participants during Round 2 are marked by an asterisk (*).*

*Outcomes reaching the pre-defined criteria for consensus during each round were taken forward to the consensus meeting and not scored again in subsequent rounds. Others were re-presented in later rounds for further consideration.*

*NR: Not rated (i.e. due to reaching consensus in earlier rounds); R1: Round 1; R2: Round 2; R3: Round 3*

**Table S3: Data for Delphi attrition bias and consensus meeting sampling bias**

| **Outcome** | **Delphi Attrition Bias (Summary scores)** | | | **Consensus Event Sampling Bias (Summary scores)** | | |
| --- | --- | --- | --- | --- | --- | --- |
|  | **R2_Participants**  **(n=123)** | **R2 Non-participants**  **(n=32)** | **Difference** | **Event Participants**  **(n=15)** | **Event Non-participants**  **(n=108)** | **Difference** |
| Nutritional status | 8 | 7 | -1 | 8 | 7 | 1 |
| Frequency of bowel sounds | 4 | 5 | 1 | 6 | 5 | 1 |
| Volume of nasogastric tube aspirate | 7 | 7 | 0 | 7 | 7 | 0 |
| Complications: Renal | 7 | 6.5 | -0.5 | 8 | 6 | 2 |
| Time to tolerate fluid intake | 7 | 7 | 0 | 7 | 7 | 0 |
| Gastric emptying | 6 | 7 | 1 | 6 | 6 | 0 |
| Mobility | 7 | 7 | 0 | 7 | 7 | 0 |
| Incidence of abdominal pain | 7 | 7 | 0 | 7 | 6 | 1 |
| Incidence of vomiting | 8 | 7 | -1 | 8 | 7 | 1 |
| Radiological intestinal dilatation | 6 | 6.5 | 0.5 | 7 | 6 | 1 |
| Anxiety | 5 | 6 | 1 | 6 | 5 | 1 |
| Need for laxative medication | 5 | 5 | 0 | 5 | 5 | 0 |
| Incidence of nasogastric tube aspirate > 500 ml per day | 7 | 7 | 0 | 7 | 7 | 0 |
| Quantification of bowel gas | 5 | 4 | -1 | 5 | 5 | 0 |
| Time to first postoperative abdominal peristalsis | 6 | 6 | 0 | 6 | 6 | 0 |
| Incidence of satiety | 5 | 5 | 0 | 5 | 5 | 0 |
| Duration of vomiting | 7 | 7 | 0 | 7 | 7 | 0 |
| Complications: Urinary | 6 | 5 | -1 | 7 | 6 | 1 |
| Complications: Pneumonia | 7 | 6 | -1 | 7 | 7 | 0 |
| Vomiting after nasogastric tube removal | 7 | 7 | 0 | 7 | 7 | 0 |
| Time to first solid intake | 7 | 7 | 0 | 7 | 7 | 0 |
| Time to return of appetite | 6 | 6 | 0 | 6 | 6 | 0 |
| Overall fluid balance | 7 | 7 | 0 | 8 | 7 | 1 |
| Postoperative inflammatory response | 7 | 8 | 1 | 7 | 7 | 0 |
| Duration of parenteral nutrition | 8 | 7.5 | -0.5 | 8 | 7 | 1 |
| Time to first passage of flatus | 7 | 7 | 0 | 7 | 7 | 0 |
| Extent of satiety | 6 | 5 | -1 | 5 | 5 | 0 |
| Duration of belching | 5 | 5 | 0 | 5 | 5 | 0 |
| Amount of food intake per meal | 6 | 5 | -1 | 5 | 6 | -1 |
| Time to second passage of flatus | 6 | 5 | -1 | 6 | 5 | 1 |
| Cumulative frequency of flatus | 6 | 5 | -1 | 6 | 5 | 1 |
| Complications: Thrombosis or embolism | 7 | 6 | -1 | 8 | 6 | 2 |
| Complications: Organ injury or failure | 8 | 7 | -1 | 8 | 8 | 0 |
| Time to detect bowel sounds | 5 | 5 | 0 | 6 | 4 | 2 |
| Time to tolerate normal diet | 8 | 6 | -2 | 7 | 7 | 0 |
| Need for antiemetic medication | 6 | 7 | 1 | 7 | 6 | 1 |
| Complications: Abdominal infection | 8 | 7.5 | -0.5 | 7 | 8 | -1 |
| Complications: Cardiac | 7 | 6 | -1 | 7 | 6 | 1 |
| Duration of nasogastric tube placement | 8 | 8 | 0 | 7 | 7 | 0 |
| Consistency of stool | 5 | 5 | 0 | 5 | 5 | 0 |
| Extent of hunger | 5 | 5 | 0 | 6 | 5 | 1 |
| Time to first soft food | 6 | 6 | 0 | 6 | 6 | 0 |
| Frequency of stool | 6 | 5 | -1 | 6 | 6 | 0 |
| Vomiting with nasogastric tube in situ | 7 | 7 | 0 | 7 | 7 | 0 |
| Incidence of nausea | 7 | 6.5 | -0.5 | 7 | 7 | 0 |
| Incidence of abdominal swelling/distension | 6 | 7 | 1 | 6 | 6 | 0 |
| Duration of nausea | 7 | 7 | 0 | 7 | 6 | 1 |
| Complications: Respiratory | 7 | 6 | -1 | 7 | 7 | 0 |
| Complications: Peritonitis | 8 | 8 | 0 | 8 | 8 | 0 |
| Complications: Wound infection | 7 | 6.5 | -0.5 | 7 | 7 | 0 |
| Time to first passage of stool | 8 | 7 | -1 | 7 | 7 | 0 |
| Incidence of hiccups | 5 | 5.5 | 0.5 | 6 | 5 | 1 |
| Severity of abdominal swelling/distension | 7 | 6.5 | -0.5 | 7 | 6.5 | 0.5 |
| Gastrointestinal-related quality of life | 7 | 7 | 0 | 7 | 7 | 0 |
| Time to tolerate solid intake | 7 | 7 | 0 | 7 | 7 | 0 |
| Time to tolerate low-residue diet | 7 | 6.5 | -0.5 | 7 | 6 | 1 |
| Incidence of belching | 5 | 5.5 | 0.5 | 6 | 5 | 1 |
| Gastrointestinal motility | 6 | 6 | 0 | 7 | 6 | 1 |
| Time to intake of > 1000 ml fluids per day | 6 | 6 | 0 | 6 | 6 | 0 |
| Complications: Sepsis | 8 | 8 | 0 | 8 | 8 | 0 |
| Gastrointestinal transit | 6 | 6.5 | 0.5 | 6 | 6 | 0 |
| Time to first fluid intake | 7 | 6 | -1 | 7 | 7 | 0 |
|  | **Median (IQR)** | **Median (IQR)** | **Median (IQR)** | **Median (IQR)** | **Median (IQR)** | **Median (IQR)** |
| Summary Statistics | 7 (6-7) | 6.5 (5.625-7) | 0 (-1-0) | 7 (6-7) | 6 (6-7) | 0 (0-1) |

*A total of 62 outcomes which were considered during both Round 1 and Round 2 are presented above. This excludes outcomes which reached consensus (removed) and outcomes which were proposed and added during Round 1. As per the Method, attrition bias within the Delphi survey was explored by comparing Round 1 summary scores (medians) of participants who did and did not subsequently take part in Round 2. Sampling attrition within the consensus meeting was explored by comparing Round 2 summary scores of participants who did and did not take part in the meeting. All values are expressed as median with interquartile range.*

*IQR: Interquartile range; R2: Round 2*
